# Supplementary material for: Highly efficient photothermal nanoagent achieved by harvesting energy via excited-state intramolecular motion within nanoparticles
Source: Nat Commun. 2019 Feb 15;10:768. doi: 10.1038/s41467-019-08722-z (PMC6377612; doi:10.1038/s41467-019-08722-z)
Supplement: Supplementary file 1 — Supplementary Information [file 41467_2019_8722_MOESM1_ESM.pdf]

Supplementary Information

Highly efficient photothermal nanoagent achieved by harvesting energy via excited-state intramolecular motion within nanoparticles

Zhao *et al*.

Methods

General. All chemicals were commercially available and used as supplied without further purification. Deuterated solvents were purchased from J&K. TEP-B(OH)$_2$ was purchased from AIEgen Biotech Co., Ltd. Tetrahydrofuran (THF) was dried by distillation using sodium as drying agent and benzophenone as indicator. Compounds 2, 3, 4, 2TPE-PDI-C$_8$ and 2TPE-PDI-C$_{16}$ were synthesized according to the published procedures. ^{1}H and ^{13}C NMR spectra were recorded on a Bruker ARX 400 NMR spectrometer using tetramethylsilane (TMS; δ = 0) as internal reference. High-resolution mass spectra (HRMS) were obtained on a Finnigan MAT TSQ 7000 Mass Spectrometer operated in a MALDI-TOF mode. Absorption spectra were measured on a JASCO V-570 UV-vis-NIR spectrophotometer. Steady-state photoluminescence (PL) spectra were recorded on an Edinburgh FLS980 fluorescence spectrophotometer. Quantum yield was determined by a Quanta-φ integrating sphere. Particle size analyses were implemented using a ZetaPlus Potential Analyzer (Brookhaven, ZETAPLUS). Transmission electron microscopy (TEM) investigations were carried out on a JEOL-6390 instrument.

PA equation. Under low-intensity irradiation, the PA response exhibits a linear dependence with respect to the incident light intensity as described by Supplementary Equation (1)[1]:

$$PA = \varepsilon_g C_g \Gamma I \Phi_{nr} \qquad (1)$$

where ε_g is the ground-state molar extinction coefficient of the contrast agent at the incident wavelength, C_g is the ground-state concentration of dye molecules, Γ is the Grüneisen coefficient, I is the incident photon fluence, and Φ_{nr} is the quantum yield for nonradiative decay. The Grüneisen coefficient, Γ, is a constant that quantifies a medium’s ability to conduct sound efficiently that is defined by Supplementary Equation (2):

$$\Gamma = V^2 \alpha / C_p \qquad (2)$$

where V_s is the velocity of sound, α is the thermal expansion coefficient of the medium, and C_p is the specific heat of the medium at constant pressure.

Photothermal conversion efficiency calculation. Photothermal conversion efficiency (η) represents the efficiency of transducing incident absorbance to thermal energy, which could be calculated as follows, according to the literature[2–4].

From an energy balance on a system, the total energy balance is:

$$\sum_i m_i C_{p,i} \frac{dT}{dt} = Q_{in,\ np} + Q_{in,\ surr} - Q_{out} \qquad (3)$$

Where the i terms $m_i C_{p,i}$ are products of mass and heat capacity of system components. T is system temperature, and t is time.

$Q_{in,np}$ is the photothermal energy input from the agents, which can be described as:

$$Q_{in,np} = I\left(1 - 10^{(-A_\lambda)}\right)\eta \qquad (4)$$

Where I is the laser power in the photothermal experiment. A_λ is the absorbance at 808 nm.

$Q_{in,surr}$ is the heat input due to light absorption by the solvent and container, which can be described as:

$$Q_{in,surr} = Q_{Dis} = hS_{buff} \times (T_{Max} - T_{Surr})_{buffer} \qquad (5)$$

Where hS_{buff} is the parameter relevant with container and solvent (h and S represent heat transfer coefficient and surface area of the container, respectively). $T_{max,buff}$ is the maximum steady-state temperature of solvent (without agents). T_{surr} is the ambient surrounding temperature.

Q_{out} is the heat lost to the surrounding, which can be described as:

$$Q_{out} = \hbar S \times (T - T_{surr}) \qquad (6)$$

The hS and hS_{buff} can be determined by measuring the rate of temperature decrease after removing the light source.

At the maximum steady-state temperature, equation (3) equals to 0 and we can obtain:

$$Q_{in,np} + Q_{in,surr} = I\left(1 - 10^{(-A_\lambda)}\right)\eta + Q_{dis} = Q_{out} = \hbar S(T_{max} - T_{surr}) \qquad (7)$$

Where T_{max} is the maximum steady-state temperature of nanoparticles. As a consequence, η is determined by:

$$\eta = \frac{\hbar S(T_{max} - T_{surr}) - Q_{dis}}{I\left(1 - 10^{(-A_\lambda)}\right)} \qquad (8)$$

Synthesis.

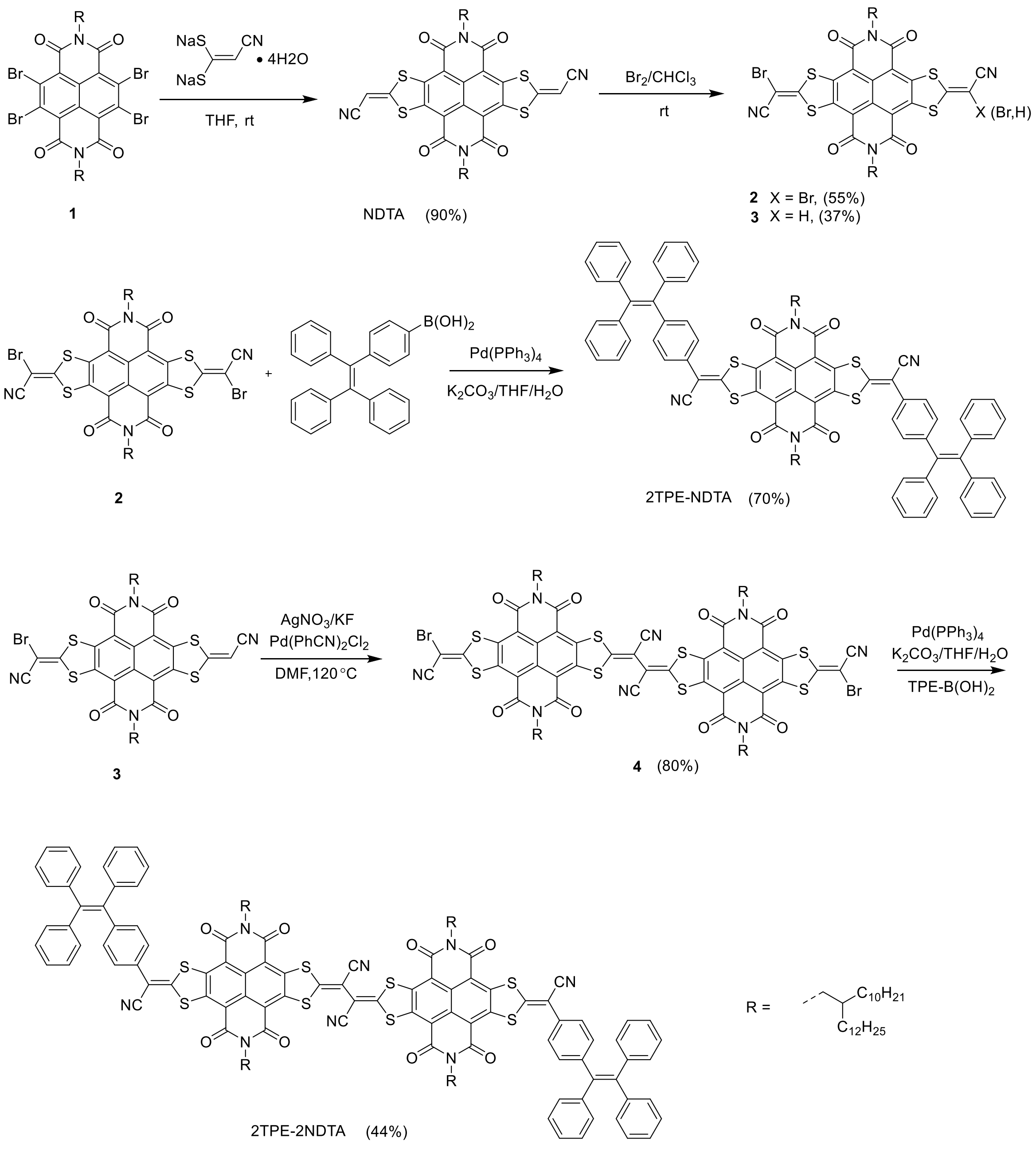

Supplementary Figure 1. The synthesis of compounds 2TPE-NDTA and 2TPE-2NDTA. Compounds **2**, **3** and **4** were synthesized according to the literature method[5,6]. Compounds 2TPE-PDI-C_6 and 2TPE-PDI-C_{16} were synthesized according to the literature method[7].

Synthesis of 2 and 3. Compound NDTA (117 mg, 0.1 mmol) and Br_2 (160 mg, 0.11 mmol), were dissolved in chloroform (8 mL) under atmosphere and reacted at room temperature for 0.5 h. The reaction was terminated by adding water and extracted with dichloromethane and

then purified by silica-gel column chromatography, affording compounds **2** and **3** with 73 mg and 46 mg, respectively. yield: 55% for **2** and 37% for **3**.

Compound 2. ^{1}H NMR (400 MHz, $CDCl_3$, 25 °C), δ (ppm): 4.17 (s, 4H), 2.04 (br, 2H), 1.38–1.22 (br, 80H), 0.87–0.84 (m, 12H). ^{13}C NMR (100 MHz, $CDCl_3$, 25 °C), δ (ppm): 162.0, 161.9, 161.6, 147.5, 147.4, 145.4, 145.2, 125.0, 124.7, 124.5, 116.5, 116.1, 114.1, 71.2, 46.2, 36.4, 32.0, 31.5 30.1, 29.7, 29.4, 26.3, 22.7, 14.1.

Compound 3. ^{1}H NMR (400 MHz, $CDCl_3$, 25 °C), δ (ppm): 5.67 (s, 1H), 4.15 (s, 4H) 1.99 (br, 2H), 1.36–1.21 (br, 80H), 0.88–0.84 (m, 12H). ^{13}C NMR (100 MHz, $CDCl_3$, 25 °C), δ (ppm): 164.5, 162.3, 162.1, 161.8, 147.5, 147.4, 147.3, 147.2, 146.9, 146.8, 144.7, 125.02, 124.8, 124.7, 116.6, 116.2, 115.6, 114.3, 85.8, 71.2, 46.2, 46.1, 36.6, 36.5, 32.0, 31.6, 31.5, 30.2, 30.1, 29.8, 29.7, 29.5, 26.5, 26.4, 22.8, 14.2.

Synthesis of 4. To a solution of compound **3** (200 mg, 0.16 mmol) in anhydrous dimethylformamide (15 mL) and dimethyl sulfoxide (3 mL), $Pd(PhCN)_2Cl_2$ (3.1 mg, 0.008 mmol), $AgNO_3$ (81.6 mg, 0.48 mmol) and KF (27.8 mg, 0.48 mmol) were added successively under N_2. The reaction mixture was stirred at 120°C for 8h under N_2. After cooling to room temperature, saturated NH_4Cl (aq) was added to the mixture and the precipitated product was filtered and collected. The crude product was purified by column chromatography using Hex/DCM (2/3, v/v) to afford pure **4** (159 mg) in a yield of 80%. MS (MALDI-TOF): *m*/*z*: [M]+ calcd for $C_{136}H_{196}Br_2N_8O_8S_8$, 2487.4; found, 2487.3. Elemental analysis: calcd, C: 65.67%, H: 7.94%, N: 4.50%; found, C: 65.39%, H: 7.82%, N: 4.31%.

Synthesis of 2TPE-NDTA. To a solution of compound **2** (150 mg, 0.11mmol), TPE-$B(OH)_2$ (213 mg, 0.57 mmol) and K_2CO_3 (125 mg, 0.91 mmol) in THF (10 mL) and H_2O (4 mL),

$Pd(PPh_3)_4$ (13.1 mg, 0.011 mmol) was added under N_2 protection. The mixture was stirred overnight at 100°C under N_2, after which the mixture was extracted with CH_2Cl_2 and the organic solvent was removed under reduced pressure. The crude product was purified by column chromatography to afford pure 2TPE-NDTA (140 mg) in a yield of 70% (Melting point: 269−270 °C).

^1H NMR (400 MHz, $CDCl_3$, 25 °C), *δ* (ppm): 7.43–7.41 (d, 4H), 7.18−7.17 (m, 36H), 7.14−7.07 (m, 20H), 4.18 (s, 2H), 4.16 (s, 2H), 2.03 (br, 2H), 1.37−1.21 (br, 80H), 0.88−0.83 (m, 12H). ^{13}C NMR (100 MHz, $CDCl_3$, 25 °C), *δ* (ppm): 162.3, 162.2, 157.2, 147.4, 147.3, 146.0, 145.9, 145.1, 143.5, 143.3, 143.2, 142.6, 139.9, 132.4, 131.6, 131.5, 131.4, 131.0, 128.1, 128.0, 127.8, 127.1, 126.9, 126.8, 126.7, 115.8, 115.5, 101.7, 46.1, 36.4, 32.0, 31.5, 30.2, 29.8, 29.7, 29.5, 26.5, 26.4, 22.8, 14.2. HRMS (MALDI-TOF): *m*/*z*: [M]+ calcd for $C_{120}H_{136}N_4O_4S_4$, 1824.9444; found, 1824.9493.

Synthesis of 2TPE-2NDTA. The compound **4** (150 mg, 0.06 mmol), TPE-B(OH)$_2$ (113 mg, 0.3 mmol), K_2CO_3 (66 mg, 0.48 mmol) and $Pd(PPh_3)_4$ (7 mg, 0.006 mmol) were mixed in THF (10 mL) and deoxygenated H_2O (3 mL) under N_2. The mixture was stirred overnight at 100 °C under N_2. After cooling to room temperature, the mixture was extracted with CH_2Cl_2 and the organic solvent was removed under reduced pressure. The crude product was purified by column chromatography to afford pure 2TPE-2NDTA (79 mg) in a yield of 44%. (Melting point: 299.2–301.2°C).

^1H NMR (400 MHz, $CDCl_3$, 25 °C), *δ* (ppm): 7.46−7.43 (m, 4H), 7.18−7.16 (m, 14H), 7.14−7.07 (m, 20H), 4.24 (s, 4H), 4.15 (s, 4H), 2.02 (br, 4H), 1.24−1.18 (br, H), 0.88−0.83 (m, 24H). ^{13}C NMR (100 MHz, $CDCl_3$, 25 °C), *δ* (ppm): 162.3, 162.2, 157.2, 147.4, 147.3, 146.0,

145.9, 145.1, 143.5, 143.3, 143.2, 142.6, 139.9, 132.4, 131.6, 131.5, 131.4, 131.0, 128.1, 128.0, 127.8, 127.1, 126.9, 126.8, 126.7, 115.8, 115.5, 101.7, 46.1, 36.4, 32.0, 31.5, 30.2, 29.8, 29.7, 29.5, 26.5, 26.4, 22.8, 14.2. MS (MALDI-TOF): *m/z*: [M]+ calcd for $C_{188}H_{234}N_8O_8S_8$, 2990.4; found, 2990.2.

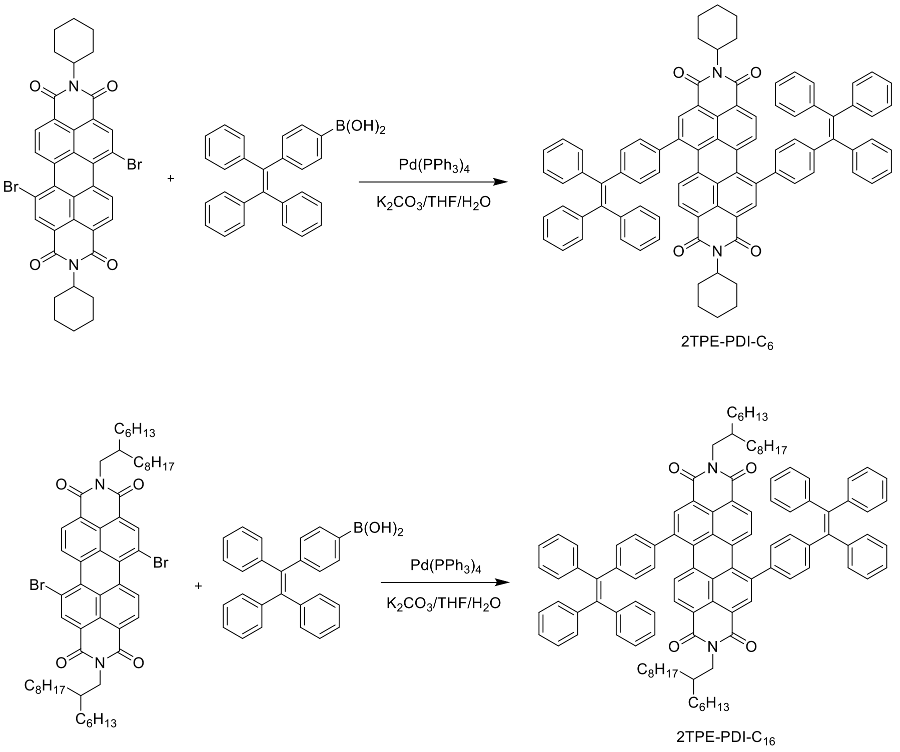

Supplementary Figure 2. The synthetic route to 2TPE-PDI-C_6 and 2TPE-PDI-C_{16}.

Synthesis of 2TPE-PDI-C_6. 2TPE-PDI-C_6 (143 mg, 0.2 mmol), TPE-$B(OH)_2$ (188 mg, 0.5 mmol), K_2CO_3 (221 mg, 1.6 mmol) and $Pd(PPh_3)_4$ (23 mg, 0.006 mmol) were mixed in THF (10 mL) and deoxygenated H_2O (3 mL) under N_2. The mixture was stirred overnight at 100 °C under N_2. After cooling to room temperature, the mixture was extracted with CH_2Cl_2 and the organic solvent was removed under reduced pressure. The crude product was by column chromatography to afford the pure 2TPE-PDI-C_6 (160 mg) in a yield of 66%.

^1H NMR (400 MHz, $CDCl_3$, 25 °C), δ (ppm): 8.52 (s, 2H), 8.17–8.15 (d, J = 8Hz, 2H), 7.80–7.78 (d, J = 8Hz, 2H), 7.19–7.07 (m, H), 5.06–5.00 (br, 2H), 2.61–2.53 (m, 4H), 1.93–1.90 (m, 4H), 1.77–1.74 (m, 6H), 1.50–1.46 (m, 4H), 1.39–1.33 (m, 2H). ^{13}C NMR (100 MHz, $CDCl_3$, 25 °C), δ (ppm): 144.5, 143.6, 143.4, 143.3, 142.0, 140.7, 140.1, 135.0, 134.4, 133.1, 132.1, 131.4, 131.3, 129.9, 129.0, 128.4, 128.0, 127.9, 127.7, 127.5, 127.0, 126.8, 126.7, 122.6, 122.2, 54.0, 29.1, 26.6, 25.5. HRMS (MALDI-TOF): m/z: [M]+ calcd for $C_{88}H_{66}N_2O_4$, 1214.5023; found, 1214.5033.

Synthesis of 2TPE-PDI-C_{16}. 2TPE-PDI-C_{16} (143 mg, 0.2 mmol), TPE-$B(OH)_2$ (188 mg, 0.5 mmol), K_2CO_3 (221 mg, 1.6 mmol) and $Pd(PPh_3)_4$ (23 mg, 0.006 mmol) were mixed in THF (10 mL) and deoxygenated H_2O (3 mL) under N_2. The mixture was stirred overnight at 100°C under N_2. After cooling to room temperature, the mixture was extracted with CH_2Cl_2 and the organic solvent was removed under reduced pressure. The crude product was purified by column chromatography to afford pure 2TPE-PDI-C_{16} (160 mg) in a yield of 66%.

^1H NMR (400 MHz, $CDCl_3$, 25 °C), δ (ppm): 8.47 (s, 1H), 8.46 (s, 1H), 8.03–8.01 (d, J = 8Hz, 2H), 7.61–7.59 (d, J = 8Hz, 2H), 7.30–7.29 (br, 6H), 7.20–7.10 (m, 32H), 4.16–4.15 (br, 4H), 1.99 (br, 2H), 1.26 (m, 50H), 0.85–0.82 (m, 12H). ^{13}C NMR (100 MHz, $CDCl_3$, 25 °C), δ (ppm): 163.7, 163.5, 144.6, 143.7, 143.4, 143.2, 142.0, 140.7, 140.0, 135.0, 134.3, 133.2, 132.0, 131.3, 129.9, 128.9, 128.8, 128.3, 128.1, 127.9, 127.8, 127.3, 127.1, 126.8, 126.7, 122.0, 121.6, 44.7, 36.7, 31.9, 31.8, 30.1, 29.8, 29.6, 29.3, 26.6, 22.7, 14.1. HRMS (MALDI-TOF): m/z: [M]+ calcd for $C_{108}H_{110}N_2O_4$, 1498.8466; found, 1498.8438.

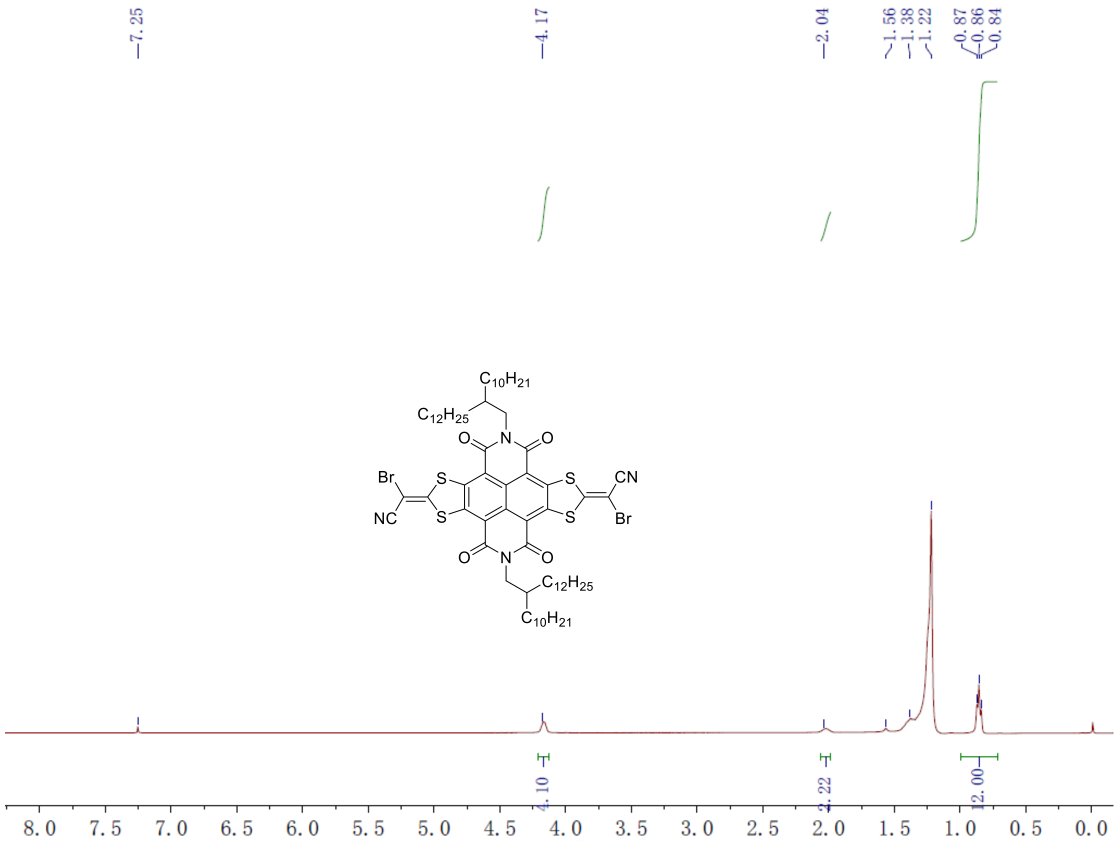

Supplementary Figure 3. ^{1}H NMR spectrum of compound **2** in $CDCl_3$ at 298 K.

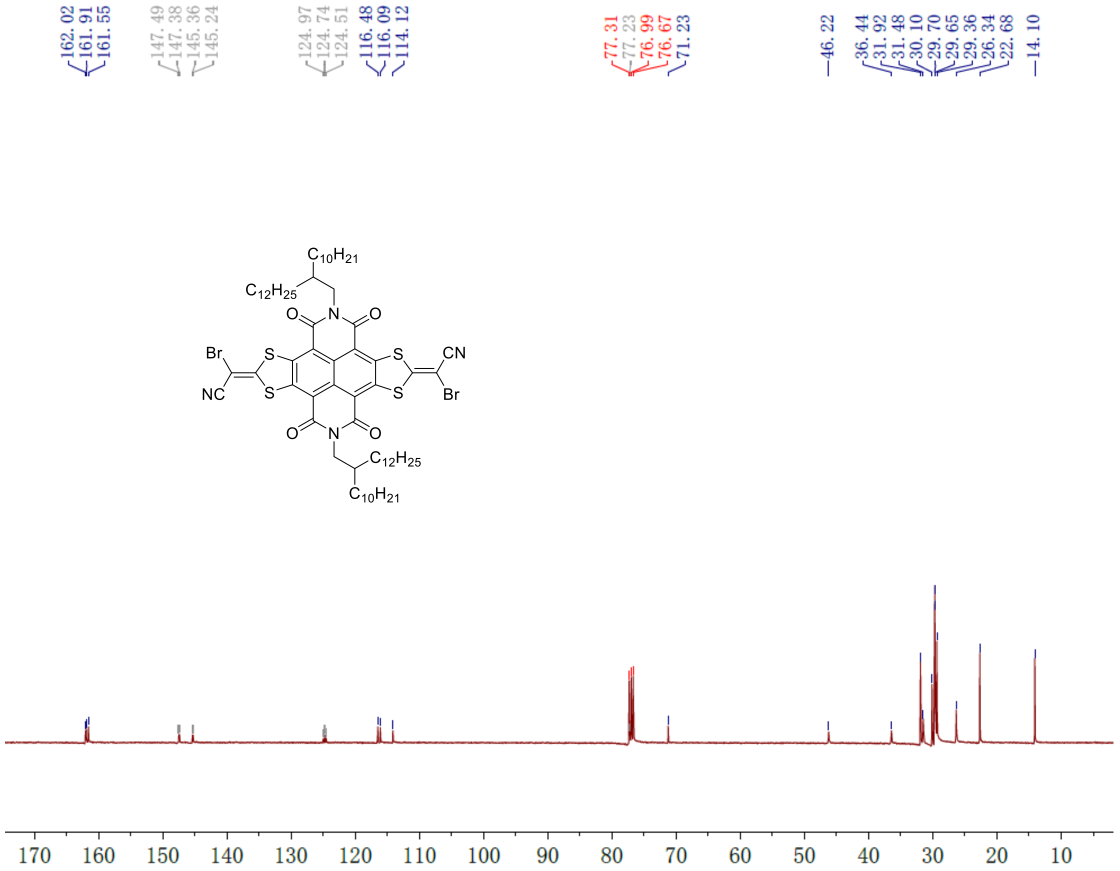

Supplementary Figure 4. ^{13}C NMR spectrum of compound **2** in $CDCl_3$ at 298 K.

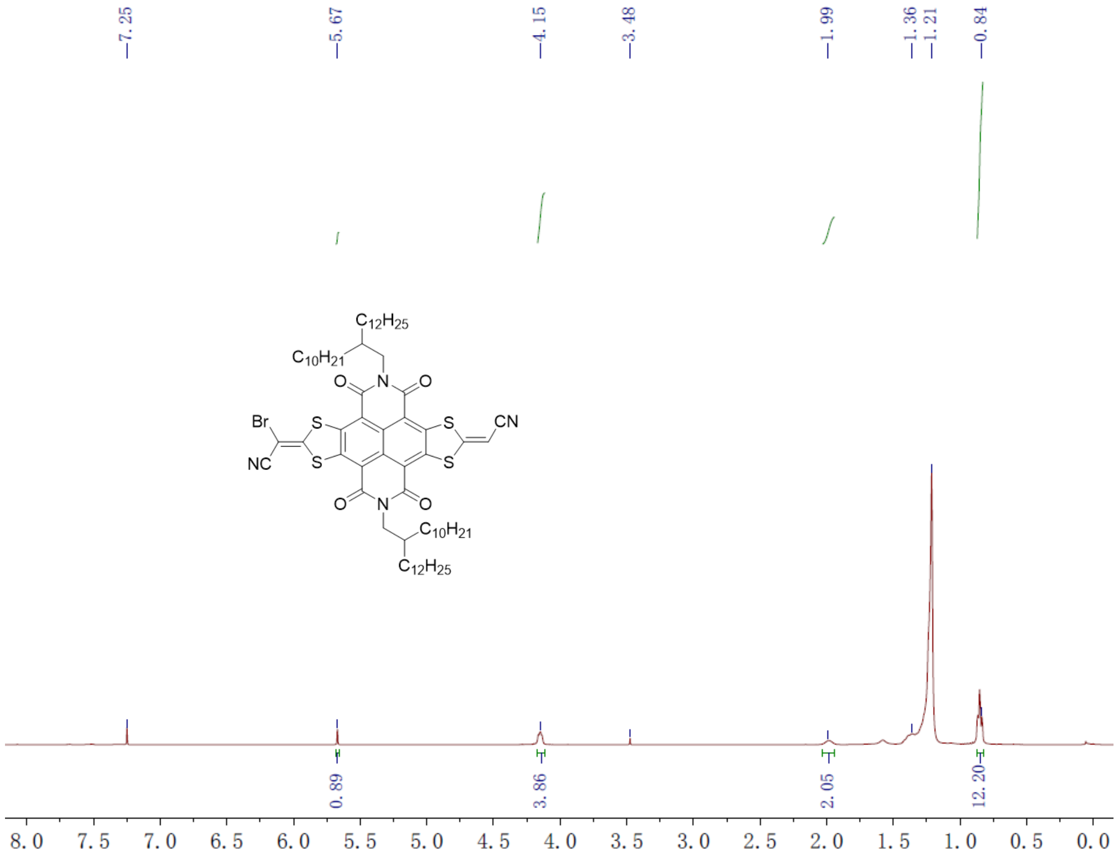

Supplementary Figure 5. ^{1}H NMR spectrum of compound **3** in $CDCl_3$ at 298 K.

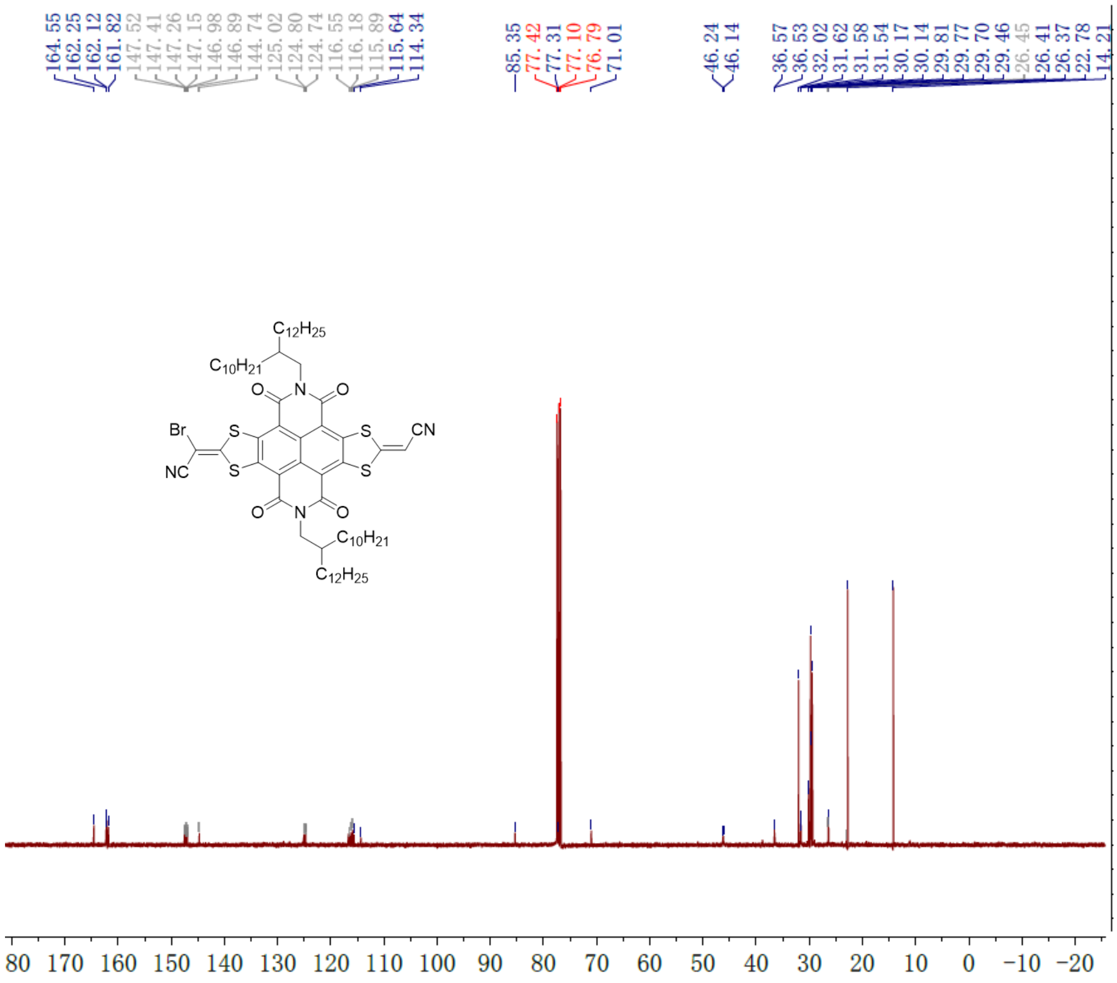

Supplementary Figure 6. ^{13}C NMR spectrum of compound **3** in $CDCl_3$ at 298 K.

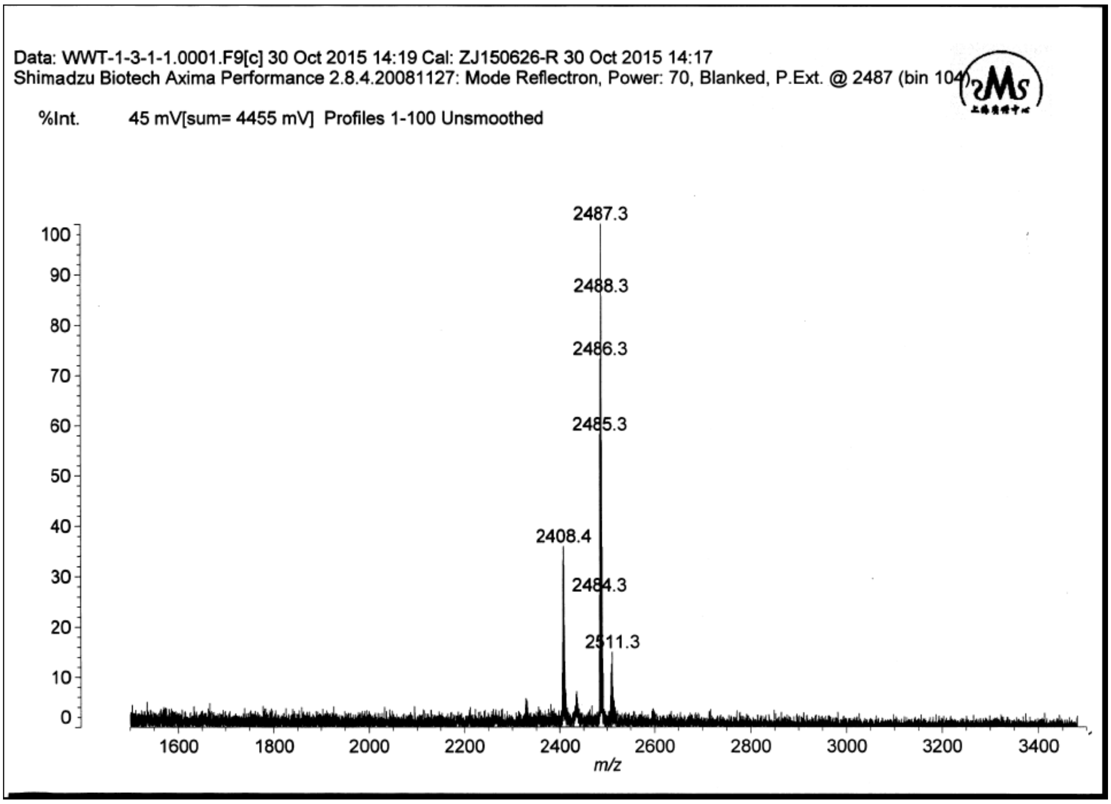

Supplementary Figure 7. High resolution mass spectrum of compound **4**.

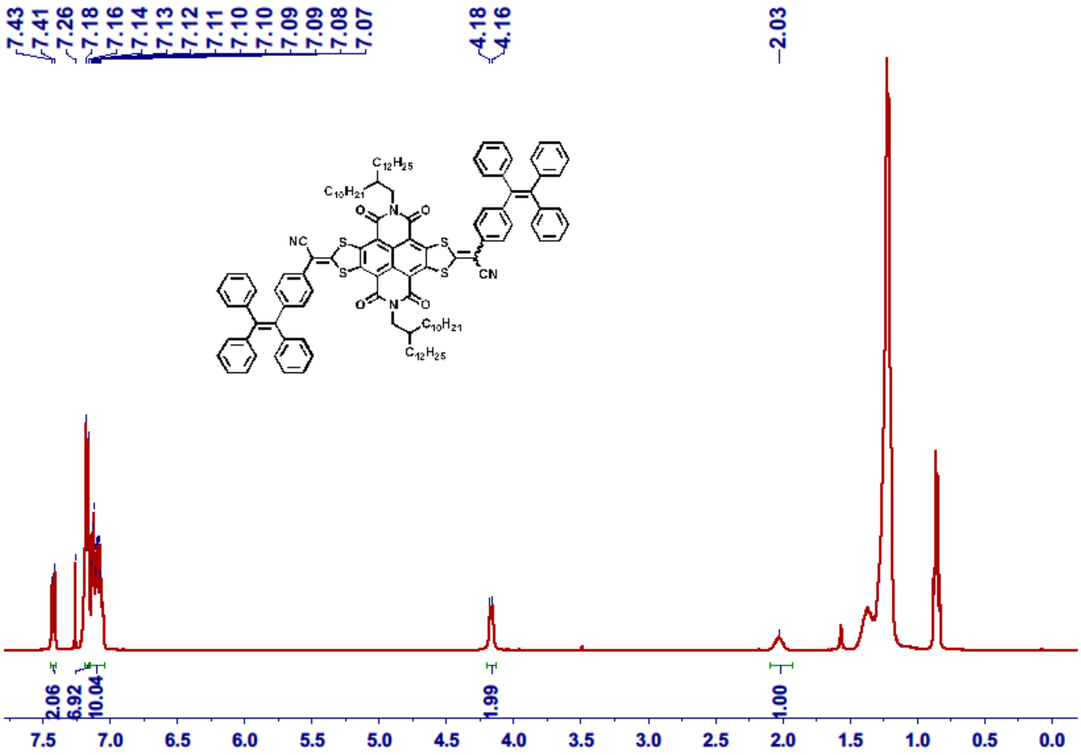

Supplementary Figure 8. ^{1}H NMR spectrum of compound **2TPE-NDTA** in $CDCl_3$ at 298 K.

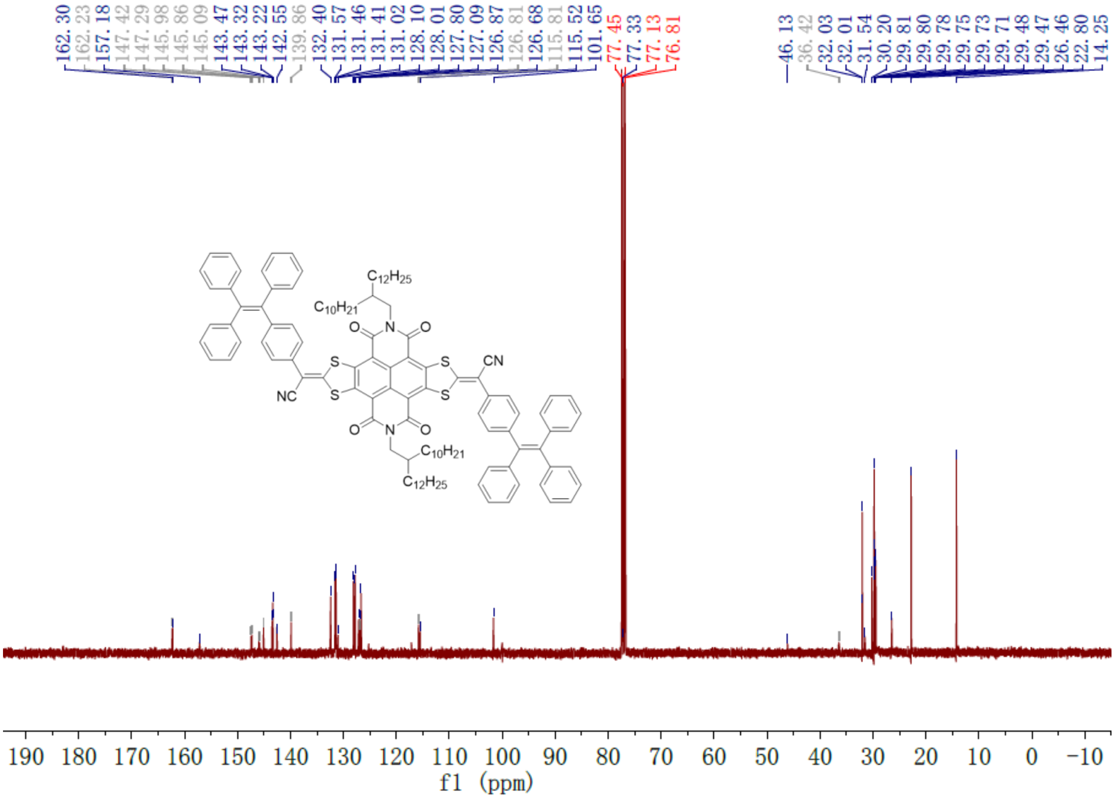

Supplementary Figure 9. ^{13}C NMR spectrum of compound **2TPE-NDTA** in $CDCl_3$ at 298 K.

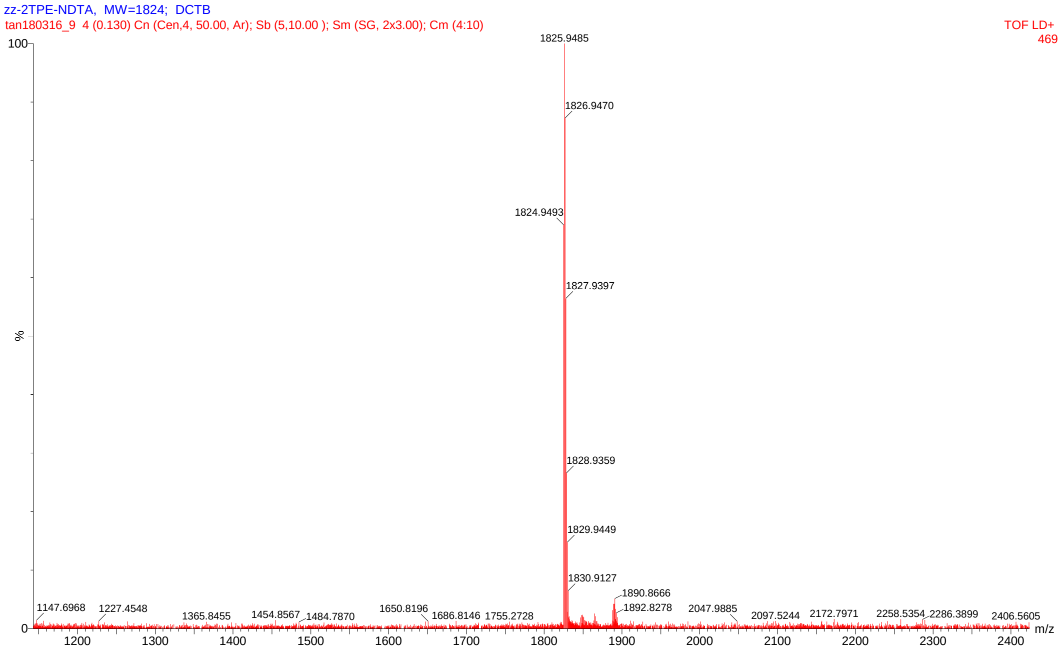

Supplementary Figure 10. High resolution mass spectrum of compound **2TPE-NDTA**.

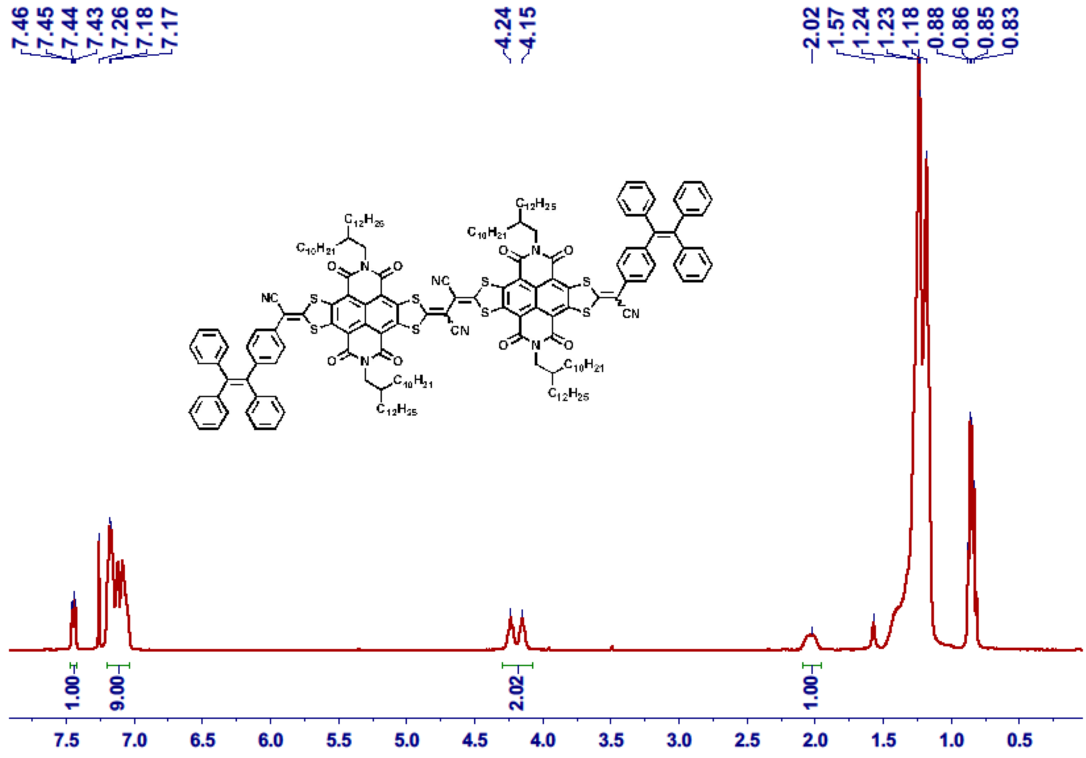

Supplementary Figure 11. ^{1}H NMR spectrum of compound **2TPE-2NDTA** in $CDCl_3$ at 298 K.

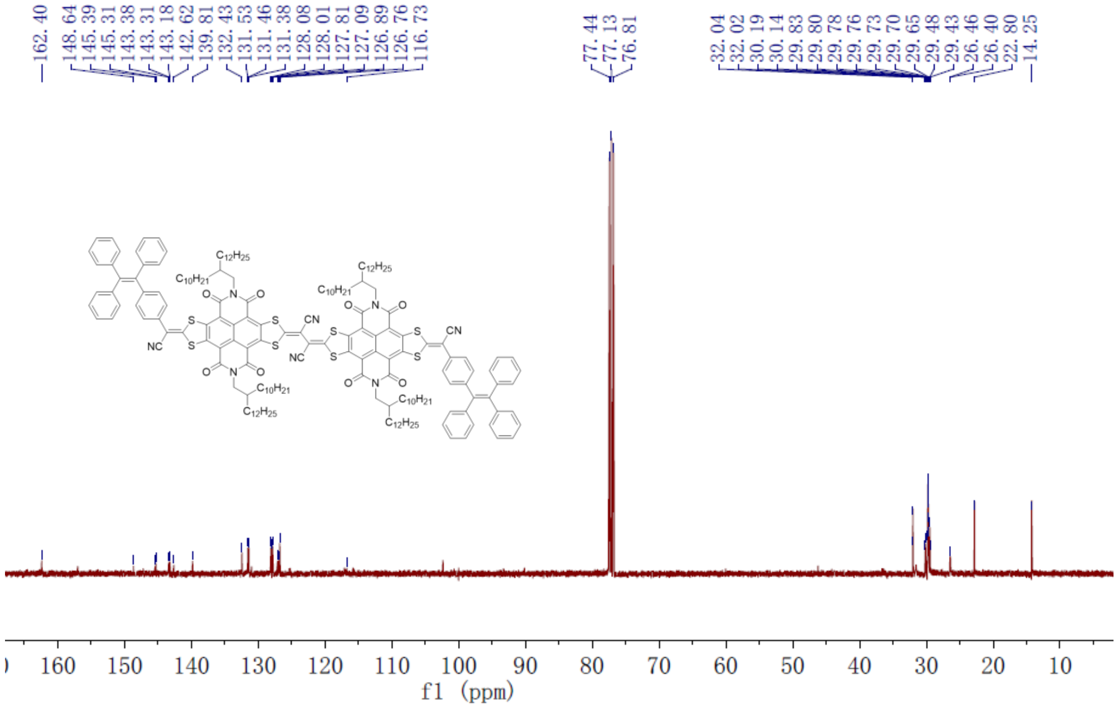

Supplementary Figure 12. ^{13}C NMR spectrum of compound **2TPE-2NDTA** in $CDCl_3$ at 298 K.

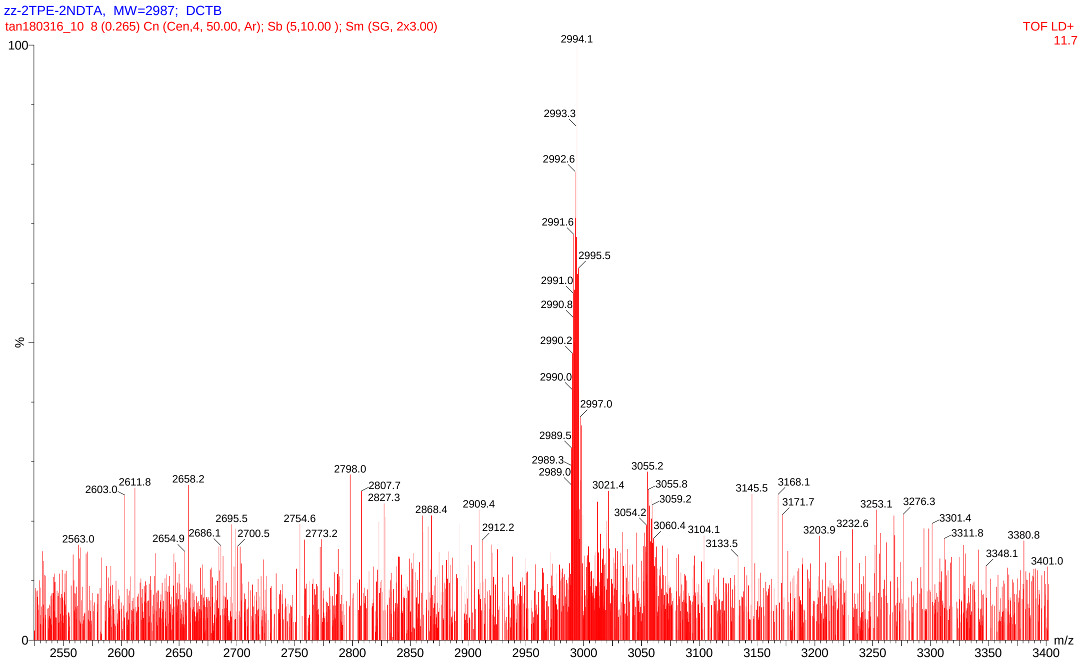

Supplementary Figure 13. Mass spectrum of compound **2TPE-2NDTA**.

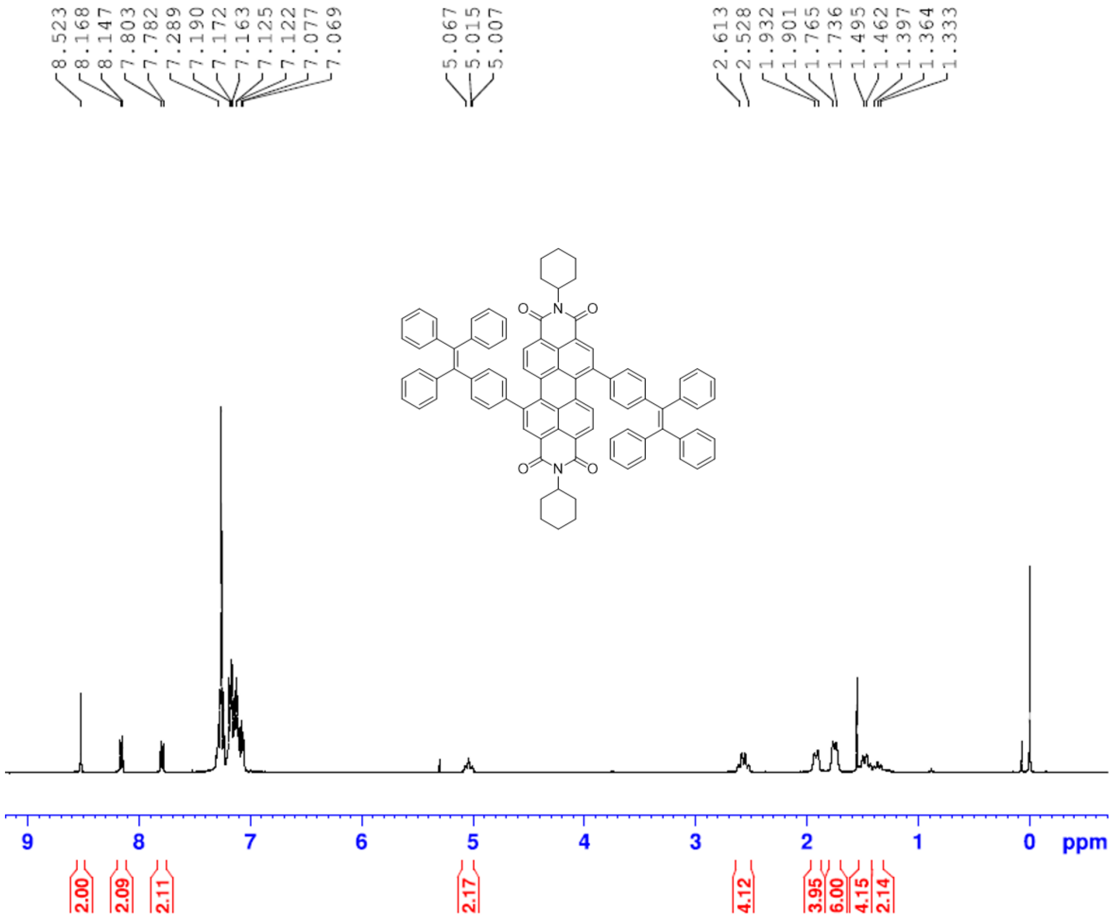

Supplementary Figure 14. ^{1}H NMR spectrum of compound **2TPE-PDI-C6** in $CDCl_3$ at 298 K.

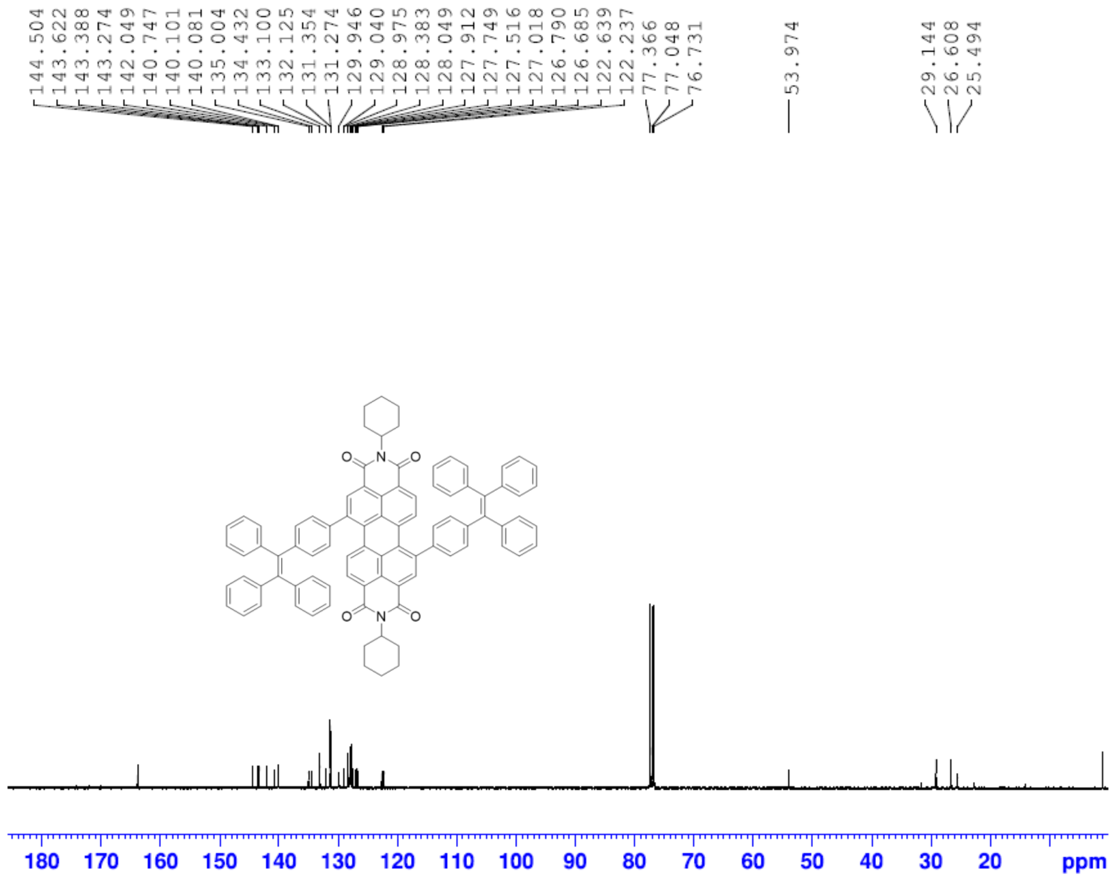

Supplementary Figure 15. ^{13}C NMR spectrum of compound **2TPE-PDI-C_6** in $CDCl_3$ at 298 K.

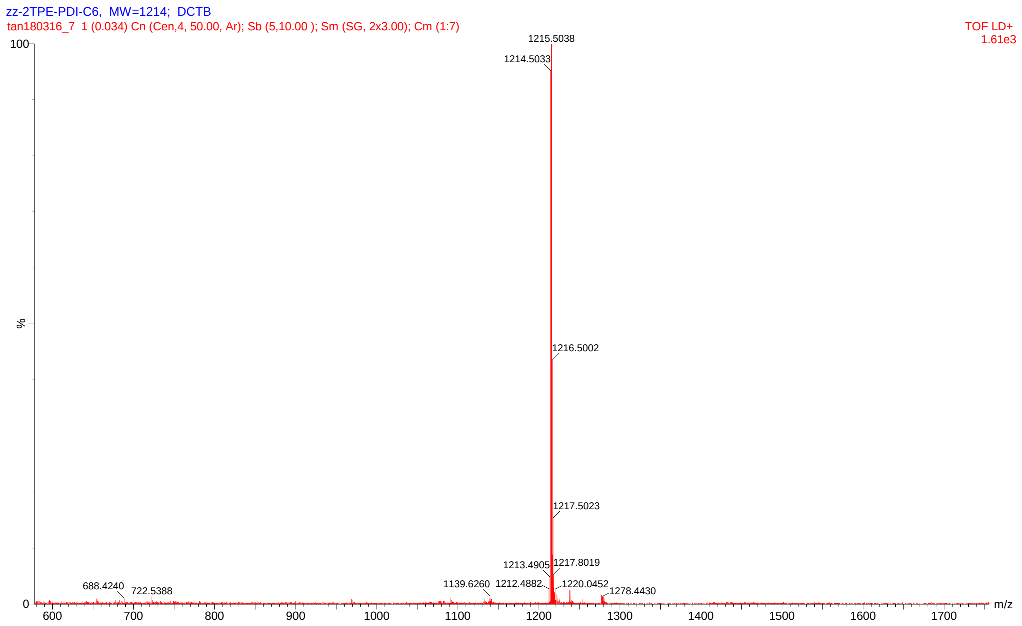

Supplementary Figure 16. High resolution mass spectrum of compound **2TPE-PDI-C$_6$**.

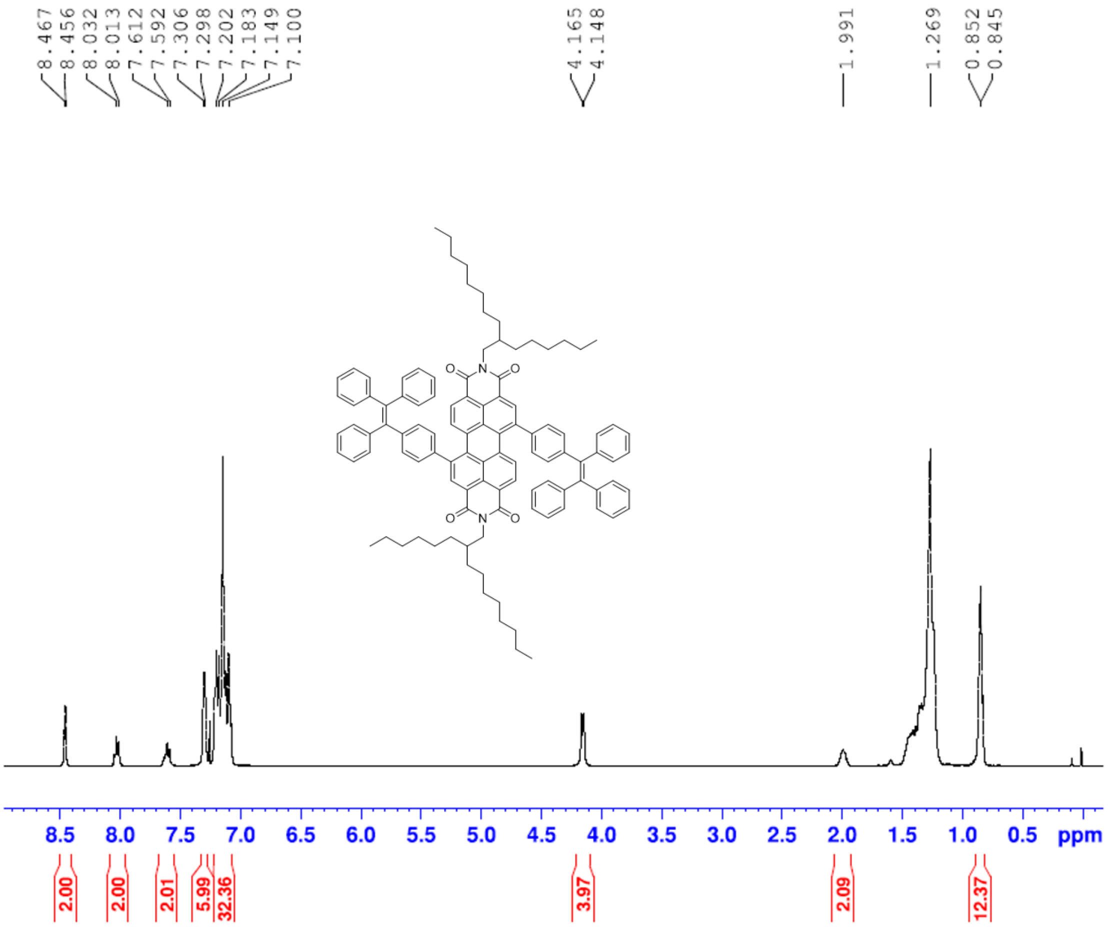

Supplementary Figure 17. ^{1}H NMR spectrum of compound **2TPE-PDI-C16** in $CDCl_3$ at 298 K.

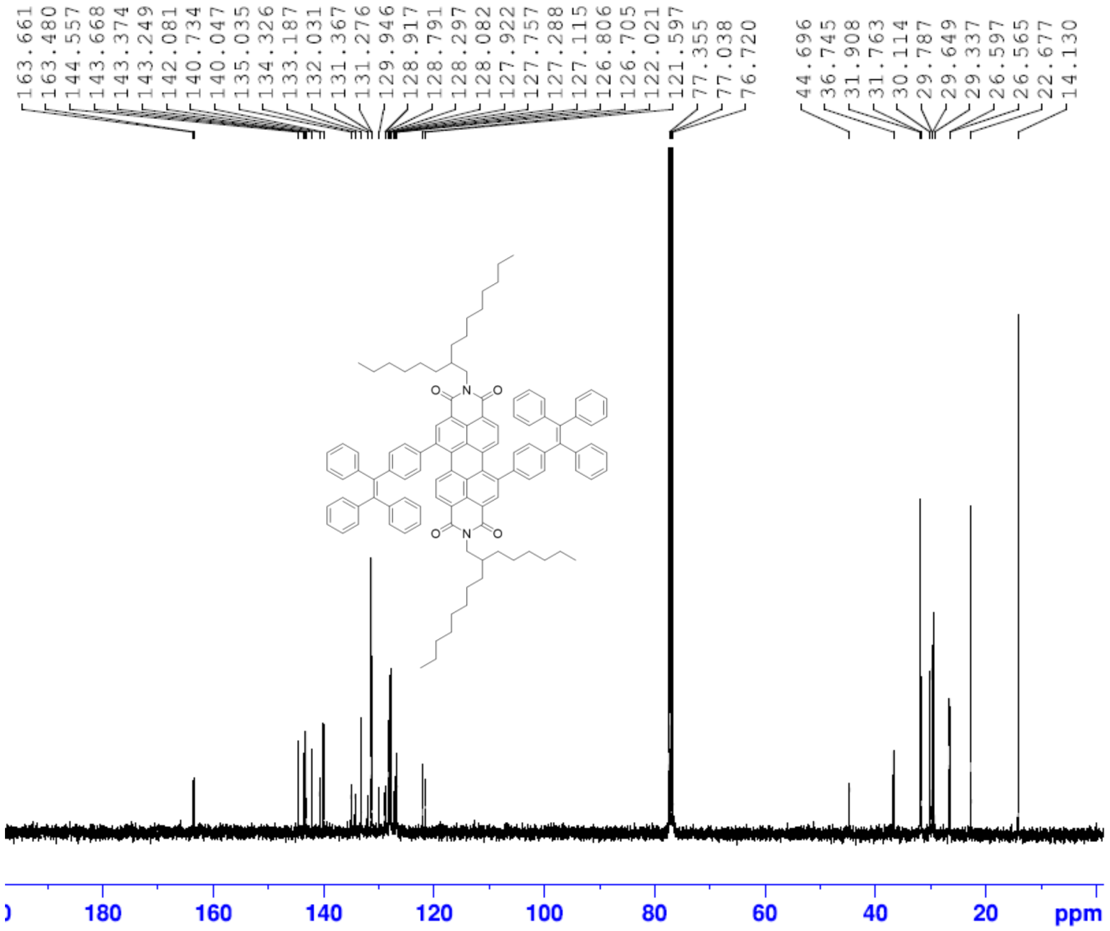

Supplementary Figure 18. ^{13}C NMR spectrum of compound **2TPE-PDI-C16** in $CDCl_3$ at 298 K.

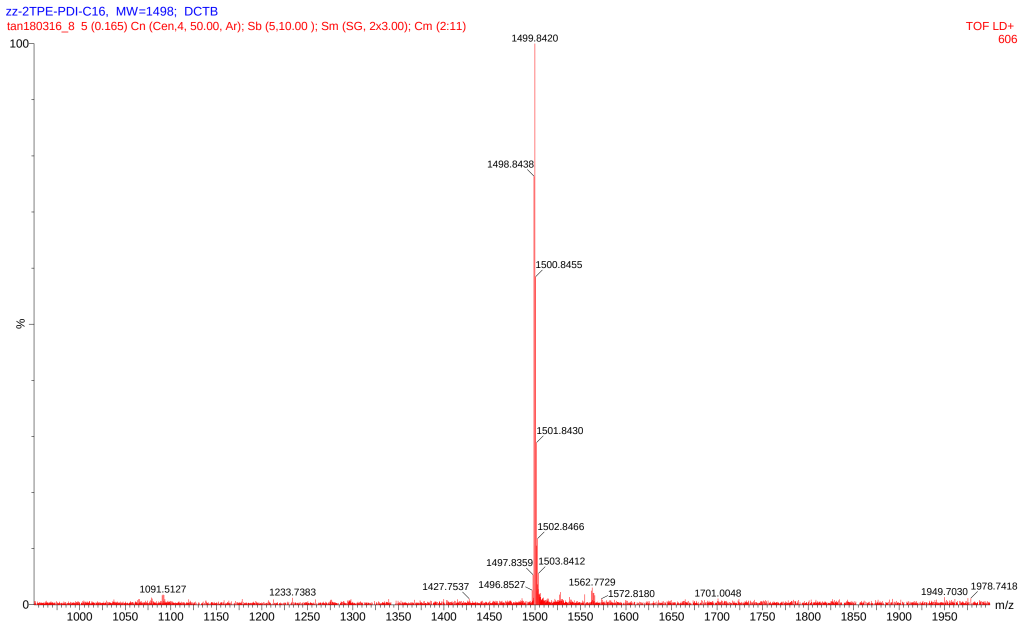

Supplementary Figure 19. High resolution mass spectrum of compound **2TPE-PDI-C$_{16}$**.

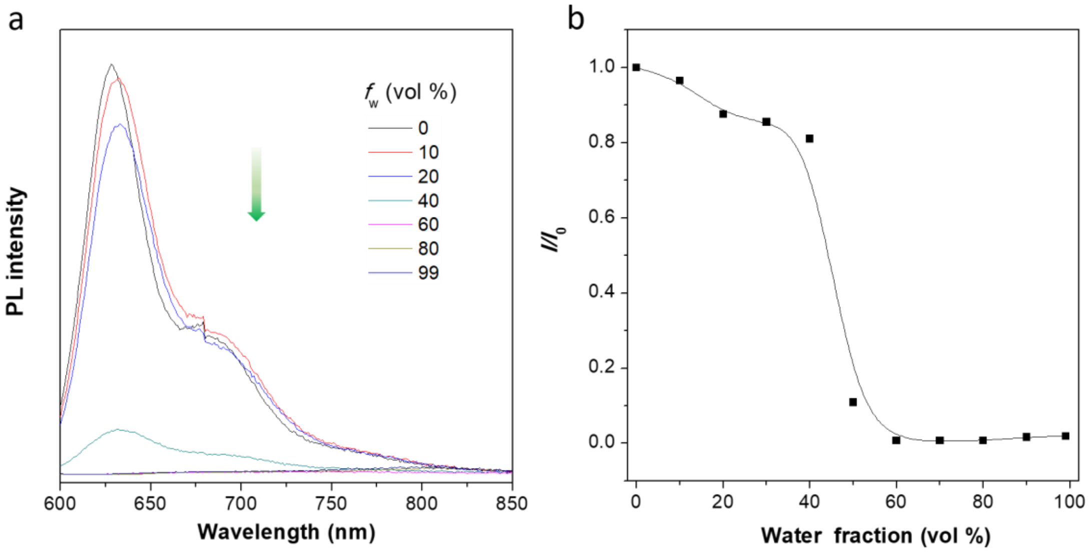

Supplementary Figure 20. **a**, PL spectra of NDTA (10 μM) in THF/Water mixtures with different water fractions (f_w). **b**, The plot of the relative emission intensity (I/I_0) versus the composition of the aqueous mixture of NDTA. I_0 = PL intensity in pure THF.

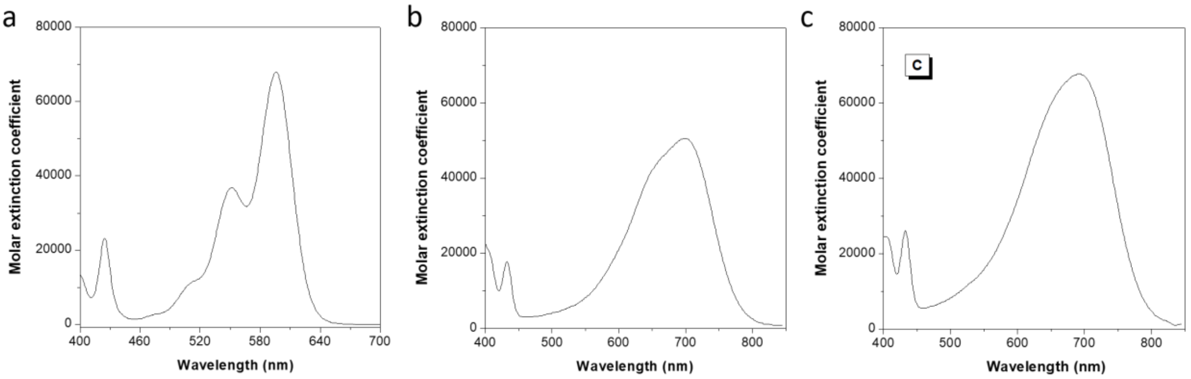

Supplementary Figure 21. **a**-**c**, UV-vis-NIR absorption spectra of compounds NDTA (**a**), 2TPE-NDTA (**b**), and 2TPE-2NDTA (**c**) in dilute THF.

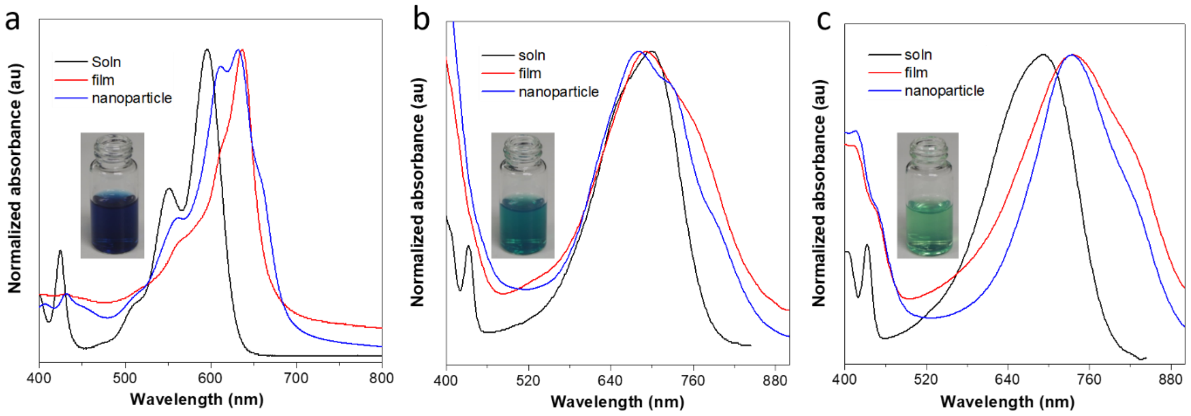

Supplementary Figure 22. **a**-**c**, UV-vis-NIR absorption spectra of NDTA (**a**), 2TPE-NDTA (**b**), and 2TPE-2NDTA (**c**) in THF solution, films and their doped organic NPs in water. Inset: photographs of the as-prepared NPs of NDTA, 2TPE-NDTA, and 2TPE-2NDTA in water.

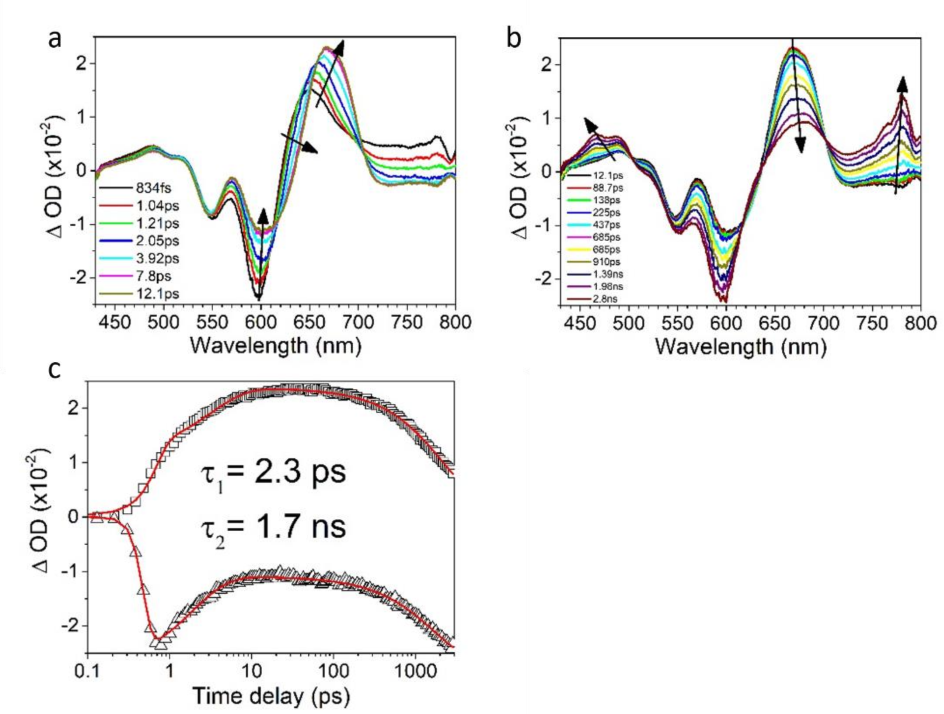

Supplementary Figure 23. a,**b**, Femtosecond transient absorption (fs-TA) spectra of NDTA at different time delays acquired after excitation at 400 nm. **c**, Kinetic traces at 670 nm (black square) and 600 nm (black triangle), solid lines (red) indicate the fitting trace to the experimental data points, and the respective fit based on a global analysis with two exponential functions.

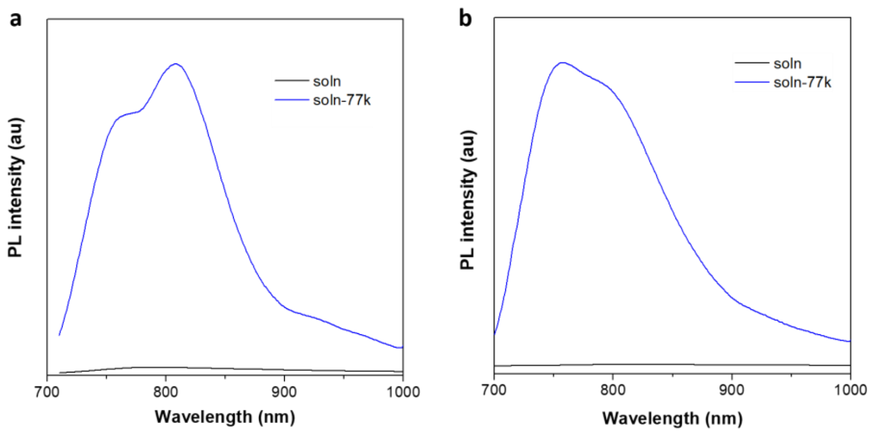

Supplementary Figure 24. **a**,**b**, PL intensity of 2TPE-NDTA (**a**), and 2TPE-2NDTA (**b**) in

THF solution at 298 K, solid at 298 K and THF solution at 77 K.

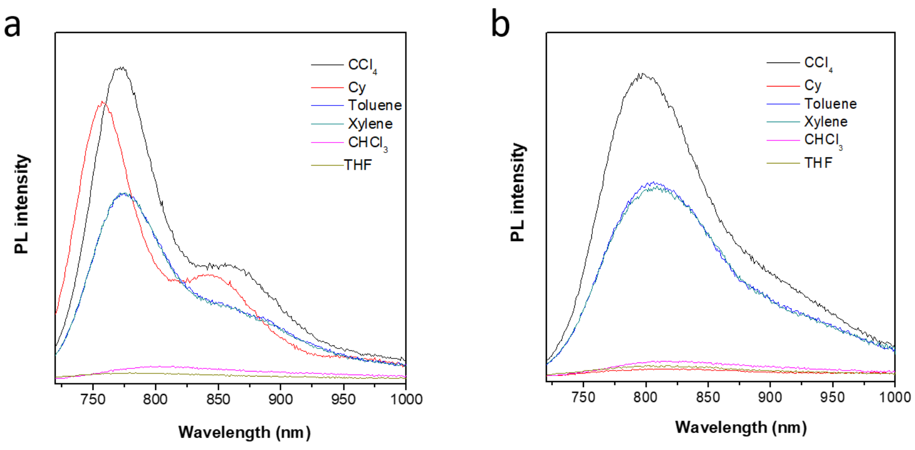

Supplementary Figure 25. **a**,**b**, PL intensities of 2TPE-NDTA (**a**), and 2TPE-2NDTA (**b**) in different solvents.

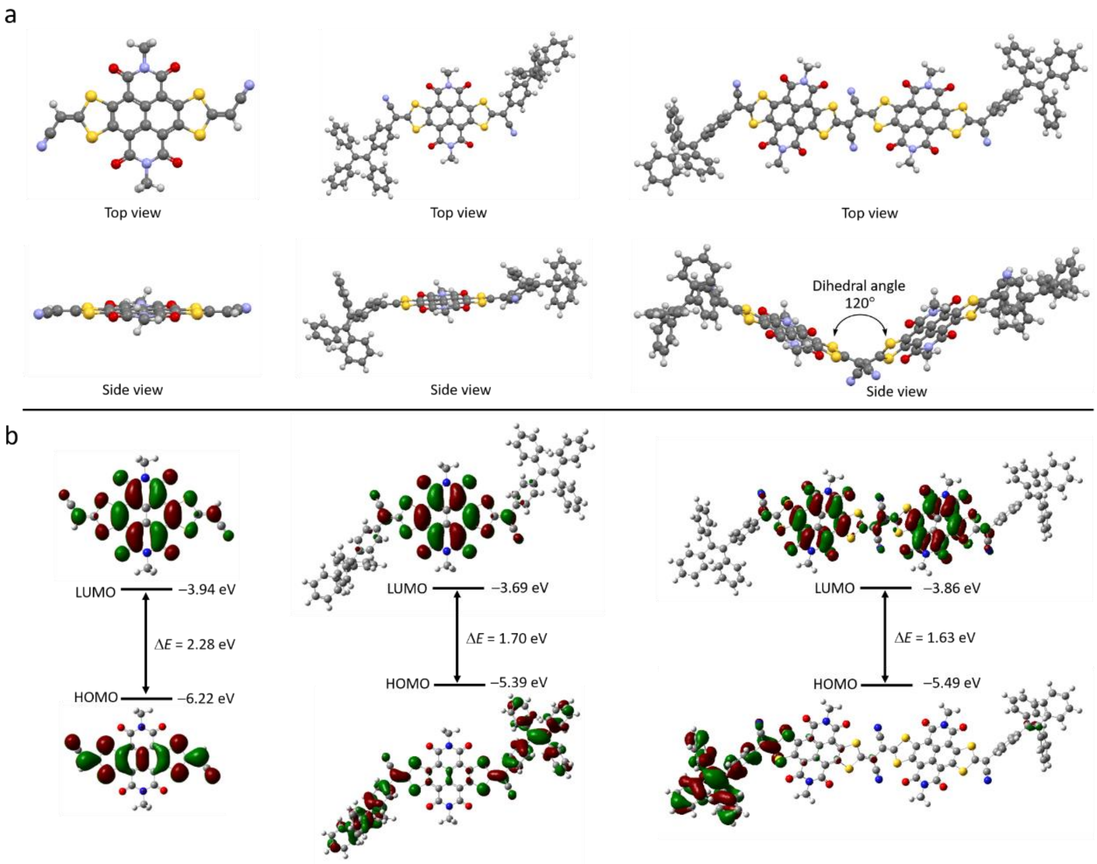

Supplementary Figure 26. **a**,**b**, The optimized molecular structure (**a**) as well as HOMO and LUMO orbital distributions, energy level, and band gaps (**b**) of NDTA, 2TPE-NDTA and 2TPE-2NDTA.

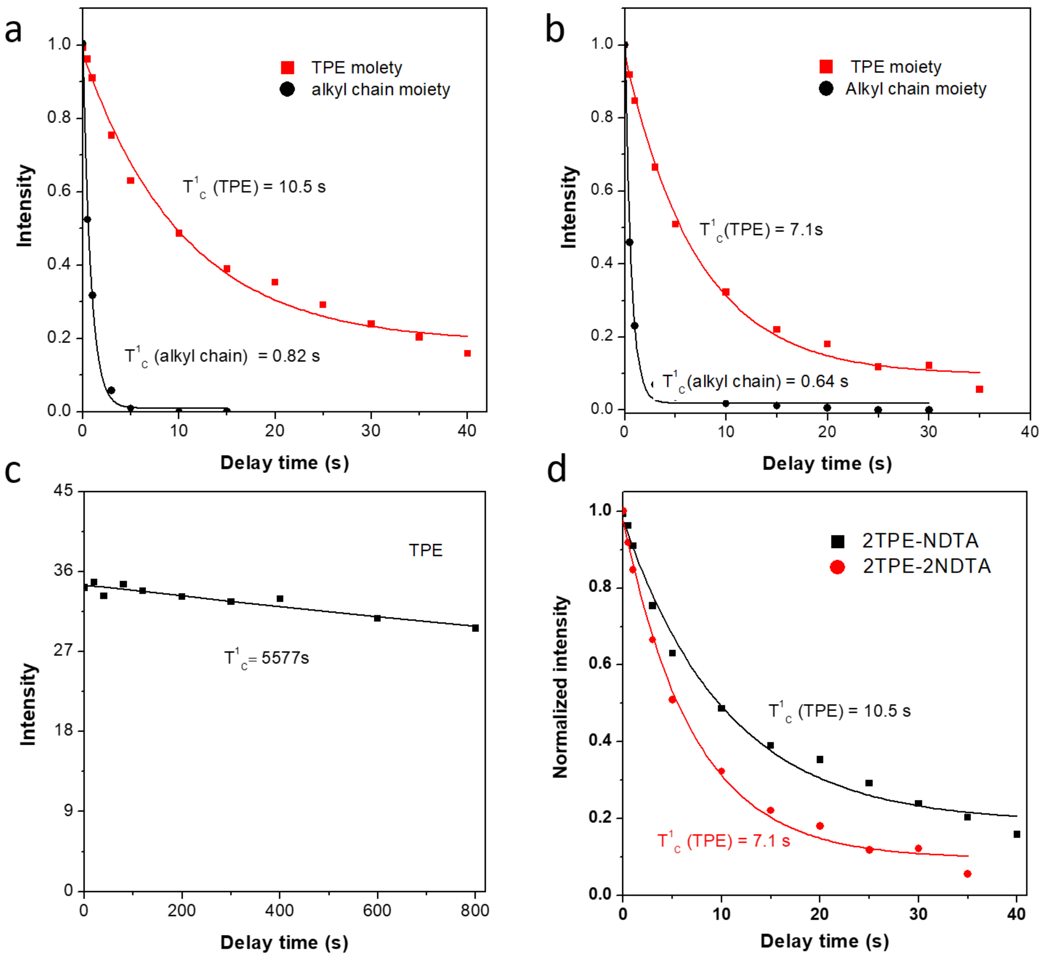

Supplementary Figure 27. **a**, The relaxation time of TPE moiety and the alkyl chain part of 2TPE-NDTA. **b**, The relaxation time of TPE moiety and the alkyl chain part of 2TPE-2NDTA. **c**, The relaxation time of pure TPE compound. **d**, Comparison of the relaxation time of the TPE part of 2TPE-NDTA and 2TPE-2NDTA.

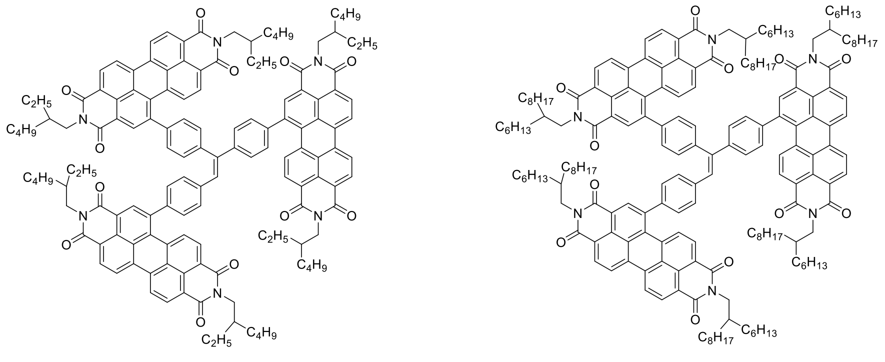

TriPE-3PDI-C_8 TriPE-3PDI-C_{16}

Supplementary Figure 28. The molecular structures of TriPE-3PDI-C_6 and TriPE-3PDI-C_{16}.

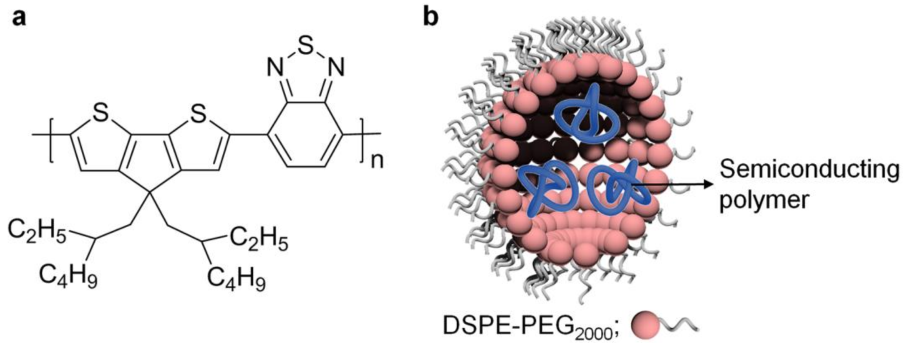

Supplementary Figure 29. **a**, Chemical structure of the semiconducting polymer: poly(cyclopentadithiophene-*alt*-benzothiadiazole). **b**, Schematic illustration of the semiconducting polymer nanoparticle (SPN).

Supplementary References

1. Borg, R. E. & Rochford, J. Molecular photoacoustic contrast agents: design principles & applications. *Photochem. Photobio.* **94**, 1175-1209. (2018)

2. Lyu, Y., Xie, C., Chechetka, S. A., Miyako, E. & Pu, K. Semiconducting polymer

nanobioconjugates for targeted photothermal activation of neurons. *J. Am. Chem. Soc.* **138**, 9049-9052 (2016).

3. Roper, D. K., Ahn, W. & Hoepfner, M. Microscale heat transfer transduced by surface plasmon resonant gold nanoparticles. *J. Phys. Chem. C* **111**, 3636-3641 (2007).
4. Hessel, C. M. et al. Copper selenide nanocrystals for photothermal therapy. *Nano Lett.* **11**, 2560-2566 (2011).
5. Zhao, Z. et al. Naphthalenediimides fused with 2-(1,3-dithiol-2-ylidene)acetonitrile: strong electron-deficient building blocks for high-performance n-type polymeric semiconductors. *ACS Macro Lett.* **3**, 1174-1177 (2014).
6. Wu, W. et al. Synthesis of largely π-extended naphthalenediimides *via* C–H activation towards highly soluble and narrow band-gap organic optoelectronic materials. *Org. Chem. Front.* **4**, 823-827 (2017).
7. Zhao, Q. et al. Tetraphenylethenyl-modified perylene bisimide: aggregation-induced red emission, electrochemical properties and ordered microstructures. *J. Mater. Chem.* **22**, 7387-7394 (2012).
